# Interobserver reproducibility of tumor uptake quantification with ^89^Zr-immuno-PET: a multicenter analysis

**DOI:** 10.1007/s00259-019-04377-6

**Published:** 2019-06-17

**Authors:** Yvonne W. S. Jauw, Frederike Bensch, Adrienne H. Brouwers, Otto S. Hoekstra, Josée M. Zijlstra, Simone Pieplenbosch, Carolien P. Schröder, Sonja Zweegman, Guus A. M. S. van Dongen, C. Willemien Menke-van der Houven van Oordt, Elisabeth G. E. de Vries, Henrica C. W. de Vet, Ronald Boellaard, Marc C. Huisman

**Affiliations:** 1 Department of Hematology, Cancer Center Amsterdam, Amsterdam UMC, Vrije Universiteit Amsterdam, De Boelelaan 1117, 1081 HV Amsterdam, The Netherlands; 2 Department of Radiology & Nuclear Medicine, Cancer Center Amsterdam, Amsterdam UMC, Vrije Universiteit Amsterdam, De Boelelaan 1117, Amsterdam, The Netherlands; 3 Department of Medical Oncology, University of Groningen, University Medical Center Groningen, Groningen, The Netherlands; 4 Department of Nuclear Medicine and Molecular Imaging, University of Groningen, University Medical Center Groningen, Groningen, The Netherlands; 5 Department of Medical Oncology, Cancer Center Amsterdam, Amsterdam UMC, Vrije Universiteit Amsterdam, De Boelelaan 1117, Amsterdam, The Netherlands; 6 Department of Epidemiology and Biostatistics, Amsterdam UMC, Vrije Universiteit Amsterdam, De Boelelaan 1117, Amsterdam, The Netherlands

**Keywords:** Monoclonal antibodies, PET, ^89^Zirconium, Immuno-PET, Reproducibility

## Abstract

**Purpose:**

In-vivo quantification of tumor uptake of 89-zirconium (^89^Zr)-labelled monoclonal antibodies (mAbs) with PET provides a potential tool in strategies to optimize tumor targeting and therapeutic efficacy. A specific challenge for ^89^Zr-immuno-PET is low tumor contrast. This is expected to result in interobserver variation in tumor delineation. Therefore, the aim of this study was to determine interobserver reproducibility of tumor uptake measures by tumor delineation on ^89^Zr-immuno-PET scans.

**Methods:**

Data were obtained from previously published clinical studies performed with ^89^Zr-rituximab, ^89^Zr-cetuximab and ^89^Zr-trastuzumab. Tumor lesions on ^89^Zr-immuno-PET were identified as focal uptake exceeding local background by a nuclear medicine physician. Three observers independently manually delineated volumes of interest (VOI). Maximum, peak and mean standardized uptake values (SUV_max_, SUV_peak_ and SUV_mean_) were used to quantify tumor uptake. Interobserver variability was expressed as the coefficient of variation (CoV). The performance of semi-automatic VOI delineation using 50% of background-corrected AC_peak_ was described.

**Results:**

In total, 103 VOI were delineated (3–6 days post injection (D3-D6)). Tumor uptake (median, interquartile range) was 9.2 (5.2–12.6), 6.9 (4.0–9.6) and 5.5 (3.3–7.8) for SUV_max_, SUV_peak_ and SUV_mean._ Interobserver variability was 0% (0–12), 0% (0–2) and 7% (5–14), respectively (*n* = 103). The success rate of the semi-automatic method was 45%. Inclusion of background was the main reason for failure of semi-automatic VOI.

**Conclusions:**

This study shows that interobserver reproducibility of tumor uptake quantification on ^89^Zr-immuno-PET was excellent for SUV_max_ and SUV_peak_ using a standardized manual procedure for tumor segmentation. Semi-automatic delineation was not robust due to limited tumor contrast.

**Electronic supplementary material:**

The online version of this article (10.1007/s00259-019-04377-6) contains supplementary material, which is available to authorized users.

## Introduction

Therapy with monoclonal antibodies (mAbs) has greatly improved the outcome of cancer patients [1]. However, treatment failure due to the biology of the disease is a substantial problem. In addition to disease-related factors, therapy-related factors have been found to be responsible [2]. There is mainly information on pharmacokinetics in blood, whereas tumor targeting is crucial for mAb efficacy. Therefore, in-vivo quantification of antibody uptake in tumors is of interest in strategies to improve the efficacy of antibody treatment (e.g. using optimized pharmacokinetic models in early drug development to improve dosing schedules). PET imaging with zirconium-89 (^89^Zr)-labelled mAbs provides a non-invasive tool to visualize and quantify mAb tumor uptake [3], providing that biodistribution of the radiolabelled mAb represents that of the total mAb dose (radiolabelled and unlabelled). The number of clinical studies on ^89^Zr-labelled mAbs, also referred to as ^89^Zr-immuno-PET, increased in recent years [4]. Sources of measurement errors (including factors such as interobserver reproducibility of tumor uptake quantification and noise induced variability) should be known to define true biological differences. A standardized method of data acquisition and tumor uptake quantification forms the basis for obtaining experimental data that will allow such an understanding.

For quantification of tumor uptake, a volume of interest (VOI) is delineated. Subsequently, a tumor uptake measure is selected to characterize tumor uptake. Maximum (max) or peak standard uptake values (SUV_max_ and SUV_peak_, respectively) provide information on a limited part of the tumor. Mean standardized uptake values (SUV_mean_) and total lesion uptake (TLU) serve to capture the entire lesion. In clinical studies, tumor uptake is quantified at a single (late) timepoint or at multiple timepoints. Additionally, quantification of tumor uptake at an early timepoint (D0) can be considered, for example, to estimate the blood volume fraction of the tumor.

**Fig. 1:**
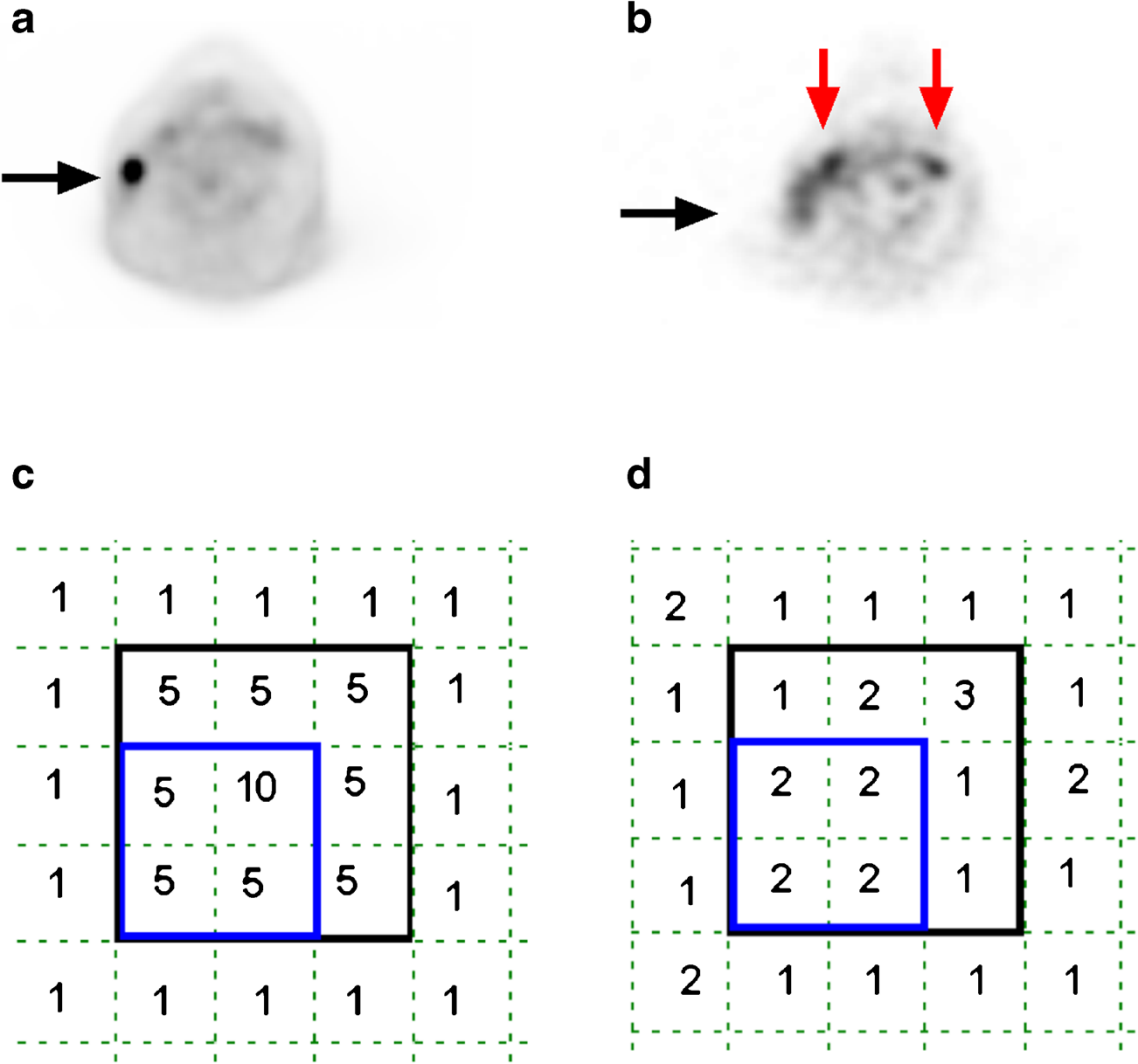
Challenges for ^89^Zr-immuno-PET tumor delineation and quantification. Example of ^18^F-FDG-PET (**a**) for a patient with a non-Hodgkin lymphoma showing intense tumor uptake (*black arrow*) and excellent contrast, while ^89^Zr-immuno-PET (**b**) with ^89^Zr-labelled-rituximab shows limited contrast for this tumor. *Red arrows* indicate uptake in blood vessels. Example of tumor delineation by two observers (observer 1 =* blue line*, observer 2 =* black line*) for ^18^F-FDG-PET (**c**) and ^89^Zr-immuno-PET (**d**). This example illustrates that excellent interobserver reproducibility (SUV_max_ = 10 for both observers) can be expected for ^18^F-FDG-PET, despite variability in tumor delineation. The limited tumor contrast for ^89^Zr-immuno-PET may result in substantial interobserver variability, even for SUV_max_ (a value of 2 and 3 for observers 1 and 2, respectively)

For imaging of mAbs, ^89^Zr is considered a suitable radioactive isotope due to its long half-life (t_1/2_ = 78.4 h), which matches the slow kinetics of large-sized proteins. Consequences of imaging with ^89^Zr are low positron abundance and relatively high radiation exposure, resulting in lower injected doses compared to ^18^F. Therefore, lower signal to noise ratios due to lower count rates may result in interobserver variability of tumor uptake quantification in ^89^Zr-immuno-PET. Other specific challenges for ^89^Zr-immuno-PET tumor delineation and quantification are relatively low, sometimes heterogeneous, tumor uptake (Fig. 1) and low (or even negative) contrast depending on tumor localization and background activity [5]. Therefore, the aim of this study was to determine interobserver reproducibility of tumor uptake values by manual delineation on ^89^Zr-immuno-PET.

## Materials and methods

### Data inclusion

For this retrospective study, ^89^Zr-immuno-PET scans with corresponding ^18^F-FDG-PET scans were collected. Data were selected from previously published clinical studies with therapeutic mAbs: ^89^Zr-rituximab in patients with B cell lymphoma ([6]; Dutch Trial Register NTR 3392), ^89^Zr-cetuximab in patients with colorectal cancer ([5]; NCT01691391) and ^89^Zr-trastuzumab in patients with breast cancer ([7]; NCT01691391). These studies had been approved by the ethics committees (Medisch Ethische Toetsingscommissie VUmc and Medisch Ethische Toetsingscommissie UMC Groningen) and all subjects signed an informed consent. Data acquisition and visual assessment of tumor uptake was done locally: from the first two studies performed at the VUmc all subjects with visible tumor uptake were included, from the last study performed at the UMCG seven subjects were selected randomly. Scan data at 1 h (D0), 72 h (D3) and 144 h (D6) post injection (p.i.) for ^89^Zr- labelled rituximab and cetuximab and at 96 h (D4) p.i. for ^89^Zr-trastuzumab were included. See Table 1 for patient characteristics and ^89^Zr-immuno-PET scan details. ^89^Zr-rituximab and ^89^Zr-cetuximab PET scans were performed on a Philips Gemini TF-64 or Ingenuity TF-128 PET-CT scanner (Philips Healthcare, The Netherlands). A Siemens Biograph mCT64 PET-CT scanner (Siemens Healthcare, The Netherlands) was used for the ^89^Zr-trastuzumab-PET scans.

**Table 1:** Patient characteristics and ^89^Zr-immuno-PET scan details

Patient	mAb	Gender	Injected dose (MBq)	^89^Zr-immuno-PET (*n* = number of VOI)			
				*D0*	D3	D4	D6
1	Rituximab	F	69.8	*1*	1	–	1
2	Rituximab	M	75.3	*22*	22	–	22
3	Rituximab	M	79.2	*2*	2	–	2
4	Rituximab	M	75.0	*0* ^a^	1	–	1
5	Rituximab	F	75.6	*6*	0	–	6
6	Cetuximab	F	36.7	*2*	2	–	2
7	Cetuximab	M	35.6	*2*	2	–	2
8	Cetuximab	F	36.2	*2*	2	–	2
9	Cetuximab	F	36.5	*1*	1	–	1
10	Cetuximab	F	35.5	*2*	0	–	2
11	Cetuximab	M	38.1	*0* ^b^	0	–	1
12	Trastuzumab	F	35.0	*–*	–	5	–
13	Trastuzumab	F	38.2	*–*	–	4	–
14	Trastuzumab	F	35.8	*–*	–	5	–
15	Trastuzumab	F	37.3	*–*	–	4	–
16	Trastuzumab	F	38.3	*–*	–	5	–
17	Trastuzumab	F	35.3	*–*	–	2	–
18	Trastuzumab	F	37.0	*–*	–	3	–
Total				*40*	103		

^a^Technical error: 1 VOI missing for patient 4

^b^No D0 scan available: 1 VOI missing for patient 11

D0 VOI were delineated on D6 and imported to the D0 scan (data marked in *italics*)

### VOI delineation

All immuno-PET scans were acquired and reconstructed to conform to recommendations for multicenter harmonization of ^89^Zr-immuno-PET [8]. Visual assessment of immuno-PET scans was performed by an experienced nuclear medicine physician (OSH for ^89^Zr-rituximab and ^89^Zr-cetuximab, AHB for ^89^Zr-trastuzumab). Tumor uptake was defined as focal uptake exceeding local background. For visually positive tumor lesions, a screenshot indicating tumor localization on immuno-PET was obtained for tumor uptake quantification. Quantitative assessment of tumor uptake for all lesions was independently performed by three observers [1 data analyst (SP), 2 physician-researchers (FB, YJ)]. Tumor delineation for all VOI was performed using the ACCURATE software tool (developed in IDL version 8.4 (Harris Geospatial Solutions, Bloomfield, USA)) [9].

The observers recorded the analysis time per tumor lesion and VOI delineation method.

#### Manual tumor delineation on immuno-PET

**Fig. 2:**
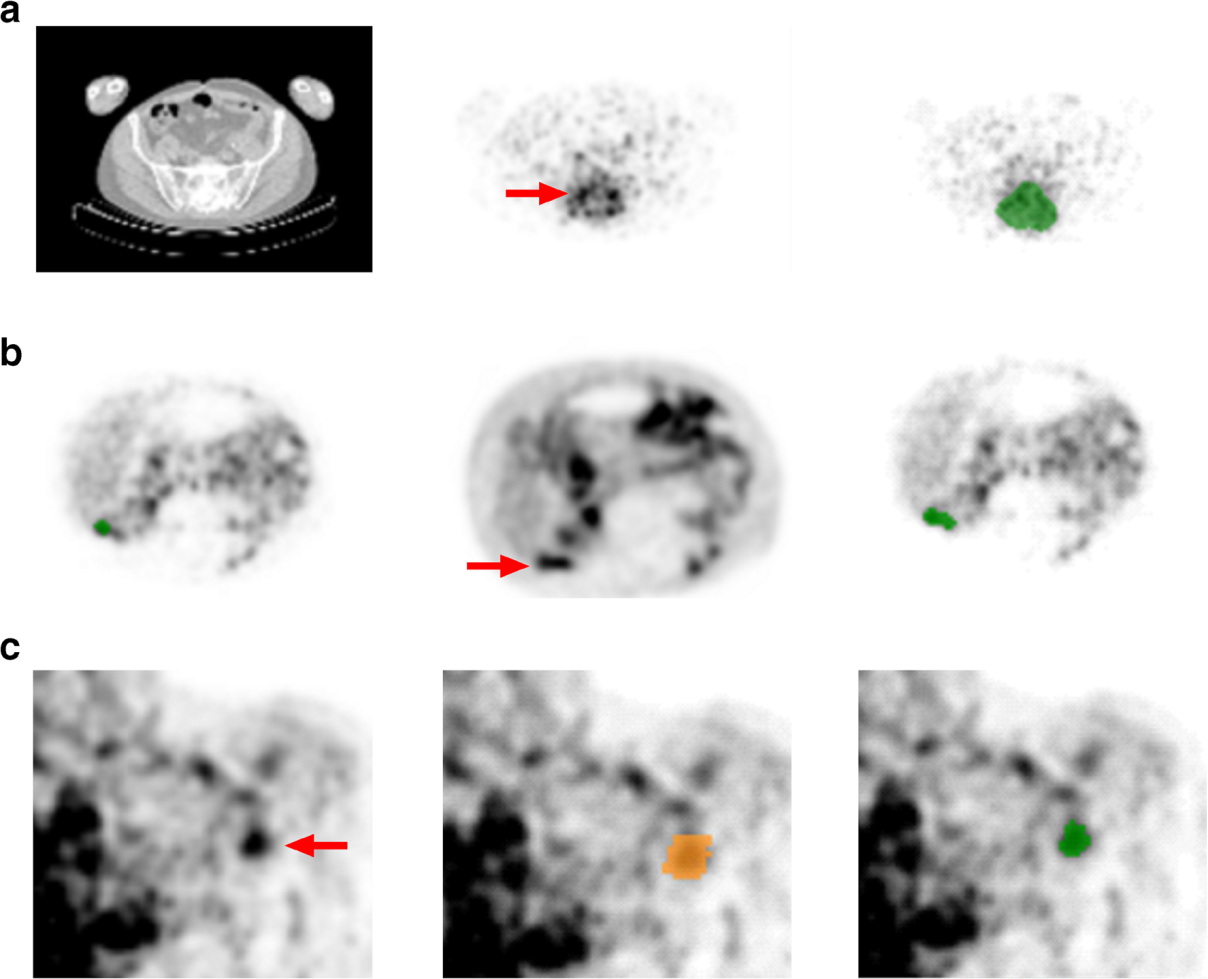
VOI delineation methods for ^89^Zr-immuno-PET. Manual tumor delineation on immuno-PET (**a**) using the low dose CT (*left panel*), attenuation corrected ^89^Zr-cetuximab-PET on D6 (*middle panel*) with tumor lesion indicated by the *red arrow* and example VOI on ^89^Zr-cetuximab-PET shown in *green* (*right panel*). Manual tumor delineation on immuno-PET after reviewing the corresponding ^18^F-FDG-PET (**b**) the original manually delineated VOI shown in *green* on the ^89^Zr-trastuzumab-PET on D4 (*left panel*), reviewing the ^18^F-FDG-PET scan with tumor lesion indicated by the *red arrow* (*middle panel*) and adapting the original VOI after reviewing the ^18^F-FDG-PET scan; the FDG adapted tumor VOI shown on ^89^Zr-trastuzumab-PET is in *green* (*right panel*). Semi-automatic delineation (**c**) with the attenuation corrected ^89^Zr-rituximab-PET on D6 (*left panel*), the mask delineated on the ^89^Zr-rituximab-PET shown in *orange* (*middle panel*) and the semi-automatic VOI (50% of AC_peak_, mask restricted) on the ^89^Zr-rituximab-PET shown in *green* (*right panel*). This semi-automatic VOI was accepted by the observer, as it contains tumor and no other structures or background

The observers manually delineated tumor VOI on the immuno-PET scans (attenuation corrected image), using the low dose CT for anatomical reference (Fig. 2a). Adjustment of the following settings was allowed: zoom, contrast and orientation (coronal/axial/sagittal). Use of a threshold (upper or lower limit) or fixed size VOI was not allowed. For ^89^Zr-rituximab and ^89^Zr-cetuximab, tumors were manually delineated on both the D3 and D6 scans, starting with the latest time point. On D0, no tumor uptake was visible, therefore the VOI delineated on D6 were imported to the D0 scan. Observers could manually adjust localization of the VOI to optimize matching of the anatomical position of the tumor lesion on the D0 scan.

For all VOI, max, peak and mean activity concentrations (AC in Bq/mL) were derived and converted to standardized uptake values (SUV), by correcting for body weight and injected dose (ID). In addition, delineated volume (mL) and TLU (defined as AC_mean_ * volume, in %ID) were obtained.

#### Manual tumor delineation on immuno-PET after viewing the ^18^F-FDG-PET

In order to support delineation of the tumor, the observers had access to the corresponding ^18^F-FDG-PET and could adapt the original manually delineated VOI if necessary (for example, by creating a smaller or larger VOI, or changing the position of the VOI) (Fig. 2b). This procedure was performed on scans with visible tumor uptake (D3, D4, D6). The number of VOIs that were adapted after viewing the ^18^F-FDG-PET was obtained.

#### Semi-automatic VOI delineation

Finally, we investigated the feasibility of a mask-restricted semi-automatic VOI delineation method. Each observer, for every tumor lesion, manually delineated a mask, which is a VOI including the tumor, excluding non-tumor structures (e.g. nearby blood vessels) on the immuno-PET scan. Subsequently, the semi-automatic VOI was generated including all voxels with a value ≥50% of background-corrected AC_peak_ within the mask (Fig. 2c). The semi-automatic isocontour was defined as 0.5 * (peak value + average background value). The background region was determined with a region growing algorithm of the tumor border, expanding three voxels away from the border of the tumor in all three dimensions [10]. The observers rated the semi-automatic VOI and accepted the VOI if it contained the tumor and no other structures or background. The number of tumor lesions for which the semi-automatic VOI was accepted by all observers was obtained.

### Eligibility criteria for VOI delineation

Quantification of lesions with low tumor uptake and/or high background uptake (e.g. lesions with low contrast and/or nearby presence of blood vessels or elevated healthy tissue uptake) is difficult, due to the intrinsically low signal to noise ratios in ^89^Zr-immuno-PET. To ensure that quantification is only reported when delineation is feasible, a method to determine eligibility for VOI delineation was explored. Criteria were selected based on the potential for incorporation in a standardized workflow for tumor identification by a nuclear medicine physician, followed by tumor delineation by a data-analyst.

When measurement variability for SUV_max_ was >0, VOI were assessed for apparent insufficient tumor contrast for manual tumor delineation.

Based on this assessment VOI were deemed ineligible for quantification, according to the following criteria:

1. A different structure was delineated by at least one observer.
2. The voxel with maximum intensity was located at the border of the VOI, of at least one observer.

Interobserver variability and reliability were analyzed for the entire group of VOI, as well as for the subset of VOI eligible for quantification.

### Interobserver reproducibility

Interobserver reproducibility for manual tumor delineation on immuno-PET was assessed by an agreement parameter (standard error of measurement (SEM)) as well as a reliability parameter (ICC; [11]). As we expected that the interobserver variability between lesions within a single patient was equal or higher than between patients, we performed a VOI-based analysis.

#### Interobserver variability

The agreement parameter reflects the measurement error due to interobserver variability [11]. For every tumor lesion, three values (value_1_, value_2_ and value_3_) were obtained from observers 1, 2 and 3, respectively. Absolute interobserver variability was calculated as:

1 $$ SEM= SD\ \left({value}_1,{value}_2,{value}_3\right), $$

where SD is the standard deviation.

SEM was calculated for each individual tumor lesion and has the same unit as the uptake measure (SUV_max_, SUV_peak_ and SUV_mean_, dimensionless; volume in mL; TLU in %ID).

Relative interobserver variability was calculated as:

2 $$ CoV= SEM/ average\ \left({value}_1,{value}_2,{value}_3\right)\ast 100, $$

where CoV (%) is the coefficient of variation.

When all observers measure the exact same tumor uptake, SEM and CoV equal 0.

Correlation of absolute and relative variability with tumor uptake was assessed. For a group of *n* VOI, the interobserver variability is given as the median (interquartile range).

#### Reliability

A reliability parameter was used to assess whether differences in tumor uptake between lesions can be distinguished, despite measurement error due to interobserver variability. A two-way random model with absolute agreement (single measure) was used to obtain the ICC and 95% confidence interval. This means that the three observers in our study were considered as a random sample of all possible observers, and the systematic differences between the observers were included in the measurement error as we were interested in absolute agreement between the observers.

Reliability, expressed as ICC, was calculated as:

3 $$ ICC={\upsigma^2}_{\mathrm{lesion}}/\left({\upsigma^2}_{\mathrm{lesion}}+{\upsigma^2}_{\mathrm{obs}}+{\upsigma^2}_{\mathrm{error}}\ \right), $$

where σ^2^_obs_ is the systematic part, and σ^2^_error_ is the random part of the measurement error, while σ^2^_lesion_ is the true variance between tumor lesions. ICC calculations were performed in SPSS, version 22.

### Statistical analysis

For comparison of interobserver variability between two groups, Wilcoxon matched-pairs signed rank test was used for paired data (e.g. SUV_mean_ on D3 and D6 for the same tumor lesions). For comparison of median CoV between multiple groups, a one-way ANOVA (non-parametric) was performed, using Friedman test with Dunn’s multiple correction to compare median CoV for paired data (SUV_mean_, SUV_max_ and SUV_peak_ for the same tumor lesions). For all statistical tests, a *p* value <0.05 was considered statistically significant. Statistical tests were performed in GraphPad Prism, version 6.02.

## Results

### VOI delineation

In total, 103 VOI were manually delineated by each observer. The number of VOI was not evenly distributed over the patients (Table 1). The range in interobserver variability (SEM for SUV_peak_) for all VOI combined was 0 to 2.3 (median 0.4, *n* = 103). The range in interobserver variability between VOI within a single patient was 0 to 2.3 (median 0.6, *n* = 22) for patient 2 (^89^Zr-rituximab at D6). Interobserver variability (SEM) at D6 for the remaining five ^89^Zr-rituximab patients ranged from 0.1 to 1.4 (median 0.3, *n* = 8).

Thus, as interobserver variability was higher within a single patient than between patients, a VOI-based analysis was performed.

Manual delineation on ^89^Zr-immuno-PET required a median time of 2 min (range 1–5 min). Viewing of the ^18^F-FDG-PET /adaption of the original VOI required an additional time of 1 min (range 1–30 min). The semi-automatic procedure required 1 min (range 1–5 min).

All observers reported difficulties to distinguish the borders of some tumor lesions on immuno-PET, especially if the tumor was in proximity to other structures with high uptake, e.g. a blood vessel. Viewing the corresponding ^18^F-FDG-PET did not resolve this issue, as the localization and borders of the tumor lesions on immuno-PET were still not fully clear when viewing both the immuno-PET and the ^18^F-FDG-PET. After viewing the corresponding ^18^F-FDG-PET, 25% of the VOI were adapted by at least one observer (Table 2).

**Table 2:** Effect of viewing the ^18^F-FDG-PET on manual tumor delineation on immuno-PET and success rate of semi-automatic delineation

Measure	^89^Zr-rituximab		^89^Zr-cetuximab		^89^Zr-trastuzumab	All
	D3	D6	D3	D6	D4	
% of VOI changed by ≥1 observer after viewing the ^18^F-FDG-PET	19%	34%	0%	10%	31%^a^	25%
% of VOI accepted after semi-automatic delineation	58%	66%	14%	30%	21%	45%

^a^No ^18^F-FDG-PET available for patient 17

Semi-automatically generated VOI were accepted by all three observers in 45% of all VOI (Table 2). Inclusion of background was the main reason for failure of semi-automatic VOI.

### Eligibility criteria for VOI delineation

Measurement variability for SUV_max_ was >0 in 25% (26/103) of the manually delineated VOI.

In 4% of the cases (4/103) a different structure (e.g. another tumor lesion) was delineated by at least one observer (2/32 (D6) for ^89^Zr-rituximab; 1/10 (D6) for ^89^Zr-cetuximab and 1/28 (D4) for ^89^Zr-trastuzumab). In 15% of the cases (15/103), the voxel with the maximum intensity was located at the border of the VOI (3/26 (D3) and 2/30 (D6) for ^89^Zr-rituximab; 5/7 (D3) and 1/9 (D6) for ^89^Zr-cetuximab and 4/27 (D4) for ^89^Zr-trastuzumab).

Application of eligibility criteria resulted in exclusion of 19 VOI, as tumor contrast was apparently insufficient for correct VOI delineation.

### Interobserver reproducibility

#### Interobserver variability

**Fig. 3:**
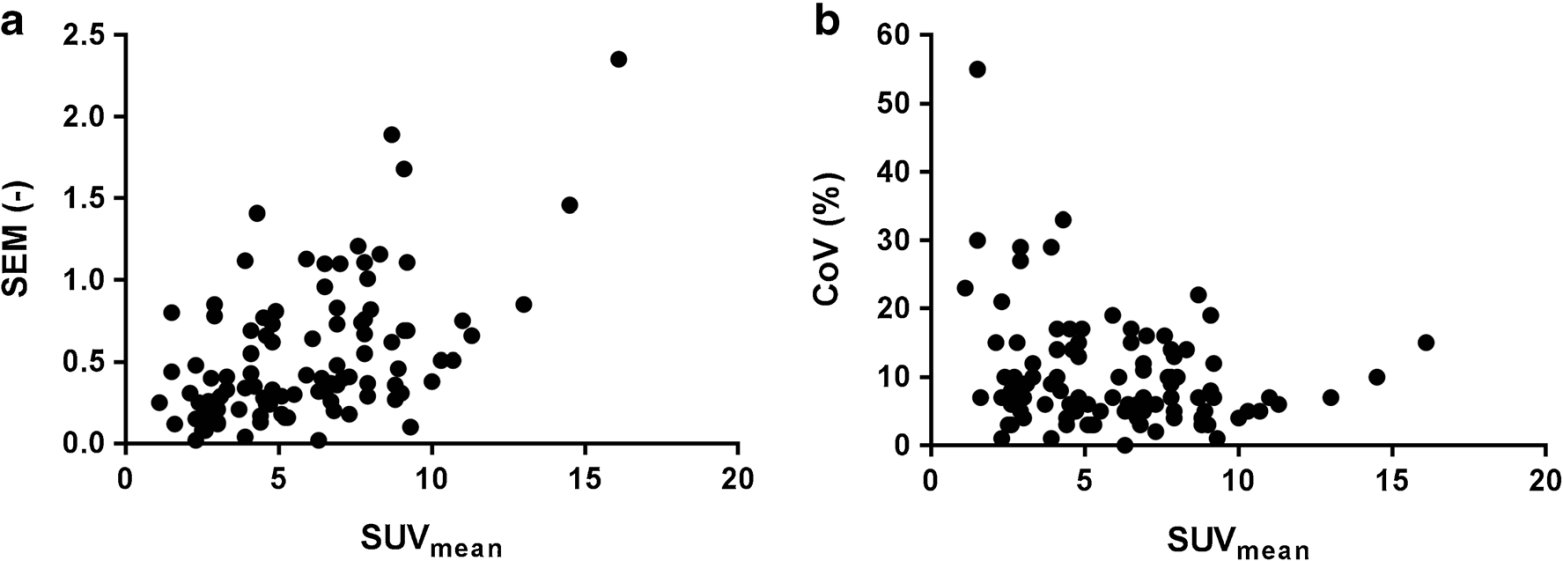
Absolute and relative interobserver variability as a function of tumor uptake. **a** Absolute variability (SEM) per individual VOI as a function of tumor uptake (SUV_mean_), Spearman correlation coefficient is 0.47 and *p* < 0.0001 (*n* = 103). **b** Relative variability (CoV) per individual VOI as a function of tumor uptake (SUV_mean_). Spearman correlation coefficient is −0.16 and *p* = 0.10 (*n* = 103). SUV_mean_ is presented as the average value for observers 1, 2 and 3

Relative interobserver variability (CoV) was not correlated with tumor uptake (SUV_mean_) (Fig. 3). Therefore, interobserver variability is reported as a relative value per individual VOI and per datagroup (e.g. timepoint, mAb) (Table 3). For all VOI combined (*n* = 103), interobserver variability was 0% (0–2) for SUV_max_, 0% (0–12) for SUV_peak_ and 7% (5–14) for SUV_mean_. Manual delineation resulted in an interobserver variability of 35% (21–49) for delineated volume and 30% (17–44) for TLU.

**Table 3:** Interobserver variability for ^89^Zr-immuno-PET

Measure		^89^Zr-rituximab		^89^Zr-cetuximab		^89^Zr-trastuzumab	All VOI combined
		D3	D6	D3	D6	D4	
SUV_max_	All	0 (0–5); *n* = 26 8.0 (5.3–11.5)	0 (0–1); *n* = 32 11.8 (5.3–17.3)	0 (0–0); *n* = 7 10.2 (9.3–13.0)	0 (0–5); *n* = 10 5.4 (3.7–8.8)	0 (0–7); *n* = 28 8.6 (5.6–12.1)	0 (0–2); *n* = 103 9.2 (5.2–12.6)
	Eligible	0 (0–5); *n* = 23 8.4 (5.3–11.4)	0 (0–0); *n* = 28 12.4 (6.1–18.0)	0 (0–0); *n* = 2 14.2(13.0–15.4)	0 (0–0); *n* = 8 4.1 (3.6–8.3)	0 (0–0); *n* = 23 9.9 (5.9–12.6)	0 (0–0); *n* = 84 9.8 (5.5–13.5)
SUV_peak_	All	0 (0–0); *n* = 26 8.7 (5.9–10.0)	0 (0–3); *n* = 32 8.5 (4.3–14.1)	0 (12–17); *n* = 7 4.2 (1.8–5.8)	0 (0–6); *n* = 10 4.4 (3.0–7.3)	18 (0–32); *n* = 28 5.6 (3.7–8.2)	0 (0–12); *n* = 103 6.9 (4.0–9.6)
	Eligible	0 (0–0); *n* = 23 8.9 (7.3–10.1)	0 (0–0); *n* = 28 9.7 (4.7–14.5)	0 (0–0); *n* = 2 5.7 (5.3–6.1)	0 (0–0); *n* = 8 3.2 (2.9–6.8)	0 (0–30); *n* = 23 6.3 (4.0–8.8)	0 (0–0); *n* = 84 7.6 (4.7–10.4)
SUV_mean_	All	6 (5–12); *n* = 26 6.8 (5.0–7.7)	7 (4–14); *n* = 32 7.0 (3.9–9.1)	12 (7–15); *n* = 7 3.3 (1.6–4.8)	10 (5–15); *n* = 10 3.8 (2.7–4.8)	7 (5–15); *n* = 28 5.0 (3.1–7.9)	7 (5–14); *n* = 103 5.5 (3.3–7.8)
	Eligible	6 (5–12); *n* = 23 6.9 (6.1–7.3)	7 (4–12); *n* = 28 7.8 (4.2–9.2)	6 (3–8); *n* = 2 4.7 (4.2–5.1)	8 (4–10); *n* = 8 3.1 (2.6–4.6)	7 (5–13); *n* = 23 5.1 (3.9–8.0)	7 (5–11); *n* = 84 6.5 (4.1–8.0)
Volume (mL)	All	26(19–47); *n* = 26 7.8 (4.2–25.0)	41(18–59); *n* = 32 7.7 (3.9–17.6)	35 (29–47); *n* = 7 3.6 (2.3–7.4)	39 (29–66); *n* = 10 4.1 (2.2–44.8)	36 (25–77); *n* = 28 4.9 (2.1–8.7)	35 (21–49); *n* = 103 6.1 (3.6–14.8)
	Eligible	23(19–35); *n* = 23 8.8 (3.8–25.6)	36(17–49); *n* = 28 7.7 (3.8–16.5)	42(35–48); *n* = 2 85.1(2.3–167.9)	35 (22–46); *n* = 8 3.2 (2.1–106)	35 (24–48); *n* = 23 5.1 (2.4–10.8)	33 (19–46); *n* = 84 6.5 (3.5–16.5)
TLU (%ID)	All	21(15–34); *n* = 26 0.06(0.03–0.13)	32(13–45); *n* = 32 0.05(0.02–0.17)	38 (34–46); *n* = 7 0.02(0.01–0.03)	32 (21–56); *n* = 10 0.01(0.01–0.26)	27 (18–70); *n* = 28 0.05 (0.01–0.08)	30 (17–44); *n* = 103 0.05 (0.02–0.12)
	Eligible	18(14–31); *n* = 23 0.07 (0.03–0.18)	27(12–42); *n* = 28 0.05 (0.02–0.15)	36(34–38); *n* = 2 0.43 (0.01–0.85)	30 (18–39); *n* = 8 0.01 (0.01–0.55)	26 (18–35); *n* = 23 0.06 (0.02–0.12)	25 (16–37); *n* = 84 0.06 (0.02–0.13)

Data is presented as interobserver variability (CoV in %) on the first line and VOI metric on the second line as median value (interquartile range)

There was no difference in interobserver variability for VOI delineated at D3 or D6 for ^89^Zr-rituximab (6 vs 8%, *p* = 0.38, *n* = 26). To obtain tumor uptake at D0 (without visible tumor contrast), a different technique was applied (importing VOI delineated at D6 to the D0 scan). Using this method, interobserver variability for SUV_mean_ at D0 was 13% (8–28) for ^89^Zr-rituximab and 10% (5–27) for ^89^Zr-cetuximab (Supplemental Table 1).

Interobserver variability did not change after viewing the corresponding ^18^F-FDG-PET (*p* = 0.62, *n* = 25 VOI adapted by at least 1 observer).

VOI eligible for quantification (*n* = 84) showed higher tumor uptake (median SUV_peak_ of 7.6 vs 3.8, *p* < 0.001) and lower interobserver variability (SUV_peak_, 0 vs 17%, *p* < 0.001) compared to ineligible VOI (*n* = 19).

##### Reliability

ICC data are presented in Table 4. For eligible VOI, ICC values for SUV_max_, SUV_peak_ and SUV_mean_ were ≥ 0.90 for ^89^Zr-rituximab (D3, D6) and ^89^Zr-cetuximab (D6). For ^89^Zr-trastuzumab, ICC values ≥ 0.82 were obtained. For volume and TLU ICC values were > 0.66 for all mAbs. In addition, ICC values for all combinations of two observers were calculated (Supplemental Table 2).

**Table 4:** Reliability of tumor uptake quantification for ^89^Zr-immuno-PET

Measure		^89^Zr-rituximab		^89^Zr-cetuximab		^89^Zr-trastuzumab
		D3	D6	D3	D6	D4
SUV_max_	All	1.00; *n* = 26 (1.00–1.00)	1.00; *n* = 32 (1.00–1.00)	0.93; *n* = 7 (0.77–0.99)	0.72; *n* = 10 (0.41–0.91)	0.97; *n* = 28 (0.95–0.99)
	Eligible	1.00; *n* = 23 (1.00–1.00)	1.00; *n* = 28 (1.00–1.00)	NA^a^	1.00; *n* = 8 (NA-NA)	0.97; *n* = 23 (0.95–0.99)
SUV_peak_	All	1.00; *n* = 26 (1.00–1.00)	1.00; *n* = 32 (1.00–1.00)	0.94; *n* = 7 (0.82–0.99)	0.75; *n* = 10 (0.46–0.92)	0.83; *n* = 28 (0.69–0.92)
	Eligible	1.00; *n* = 23 (1.00–1.00)	1.00; *n* = 28 (1.00–1.00)	NA^a^	1.00; *n* = 8 (1.00–1.00)	0.82; *n* = 23 (0.64–0.91)
SUV_mean_	All	0.92; *n* = 26 (0.84–0.96)	0.95; *n* = 32 (0.91–0.97)	0.93; *n* = 7 (0.79–0.99)	0.79; *n* = 10 (0.56–0.96)	0.94; *n* = 28 (0.89–0.97)
	Eligible	0.90; *n* = 23 (0.80–0.96)	0.94; *n* = 28 (0.90–0.97)	NA^a^	0.92; *n* = 8 (0.73–0.98)	0.93; *n* = 23 (0.86–0.97)
Volume (mL)	All	0.85; *n* = 26 (0.74–0.92)	0.12; *n* = 32 (−0.07–0.36)	0.80; *n* = 7 (0.46–0.96)	0.83; *n* = 10 (0.60–0.95)	0.67; *n* = 28 (0.48–0.82)
	Eligible	0.87; *n* = 23 (0.77–0.94)	0.72; *n* = 28 (0.55–0.85)	NA^a^	0.83; *n* = 8 (0.56–0.96)	0.66; *n* = 23 (0.45–0.82)
TLU (%ID)	All	0.90; *n* = 26 (0.83–0.95)	0.65; *n* = 32 (0.47–0.79)	0.86; *n* = 7 (0.61–0.97)	0.89; *n* = 10 (0.72–0.97)	0.71; *n* = 28 (0.54–0.84)
	Eligible	0.90; *n* = 23 (0.82–0.96)	0.83; *n* = 28 (0.72–0.91)	NA^a^	0.89; *n* = 8 (0.70–0.98)	0.70; *n* = 23 (0.51–0.85)

Data presented as ICC (95% confidence interval)

^a^*NA* ICC not available, 2 eligible VOI

## Discussion

Interobserver reproducibility for tumor uptake measures was investigated, as knowledge of measurement error is required for future clinical application of ^89^Zr-immuno-PET. Interobserver reproducibility was excellent for SUV_max_ and SUV_peak_ (variability of 0%) and very reasonable for SUV_mean_ (variability of 7%), especially considering the lower signal to noise ratios for ^89^Zr-immuno-PET compared to ^18^F-FDG-PET. For example, interobserver variability of 14% for SUV_mean_ has been reported for manual tumor delineation of pulmonary lesions on ^18^F-FDG-PET [12].

For ^89^Zr-immuno-PET, this is the first study to report interobserver reproducibility of tumor uptake measures. Several factors should be considered to determine to which extent these results are generalizable. Interobserver reproducibility was determined for three different ^89^Zr-labelled mAbs (rituximab, cetuximab and trastuzumab), at different time points (D3, D4, D6) and different injected doses (74 MBq for ^89^Zr-rituximab vs 37 MBq for ^89^Zr-trastuzumab and ^89^Zr-cetuximab). This study was not designed to assess how these factors individually impact interobserver variability. Instead, the results obtained reflect a broad range of uptake characteristics, which can be used as a general estimate of the measurement error due to interobserver variability in VOI delineation. Future, larger studies can focus on factors that influence tumor contrast (e.g. tumor localization, differences in uptake characteristics between mAbs).

Although ICC are reported, reliability is dependent on the range in tumor uptake and therefore not directly generalizable to other studies. In addition, tumor uptake and interobserver variability are influenced by the disproportionate high number of lesions in patient 2. Therefore, ICC values for this lesion-based analysis cannot be applied to determine whether we can reliably detect differences between patients.

Improved tumor contrast, in combination with a broad range in tumor uptake, is expected to result in improved interobserver reproducibility for all tumor uptake measures.

Another aspect to consider is that all observers used the same quantification software and a standardized operating procedure (no use of thresholds or fixed size VOI). Use of different software platforms without a standardized procedure may result in lower interobserver reproducibility. In addition, generalizability could be hampered if the three observers would have read the images in a systematically different way. In this study, there was no indication for such a systematic difference between the three observers.

These results suggest that interobserver agreement for SUV_mean_ is sufficient to consider this uptake measure to quantify tumor uptake in a larger tumor area (opposed to only the maximum voxel or very small sample of the tumor as defined by SUV_peak_). However, manual tumor delineation is a laborious task. As the concept of total lesion mAb uptake is of interest, the feasibility of semi-automatic VOI delineation was explored. For ^18^F-FDG-PET with perfect interobserver agreement for SUV_max_ [13] and higher tumor contrast, semi-automatic procedures are used to obtain SUV_mean_ based on a semi-automatic method (e.g. with a threshold of 0.6 of SUV_max_), total lesion glycolysis (TLG) and total metabolic tumor volume (TMTV) [14, 15]. For our datasets, the area included by the semi-automatic VOI was often too large, indicating low tumor to local background ratios, resulting in inclusion of background voxels in the semi-automatic VOI. For mAbs showing higher tumor contrast, as well as imaging with higher count statistics (due to, for example, higher injected doses or the availability of scanners with improved detection sensitivity or time of flight resolution), semi-automatic delineation may be feasible. Reduction of noise (e.g. by introduction of total body PET scanners) is the first step towards further improvement of tumor delineation procedures. Future studies into accuracy of tumor delineation should include ‘supervised’ delineation methods (semi-automatic procedures with a manual check) in which the optimal threshold is experimentally determined. If the success rate can thus be increased, this may lead to further development towards a robust automatic method, which is desired for clinical application.

As semi-automatic delineation was not feasible in our datasets, we explored eligibility criteria to improve standardization for manual tumor delineation, especially in case of limited tumor contrast.

In our study, 81% of the VOI (84 out of 103) were considered suitable for quantification. Based on these results, we recommend a two-step procedure to exclude lesions with insufficient tumor contrast for manual delineation: (1) verification of VOI delineation by a nuclear medicine physician to identify delineation of an incorrect structure due to limited tumor contrast, (2) exclusion of VOI with the voxel with the highest uptake located at the border of the VOI, indicating low tumor uptake and/or high background uptake.

These measures support optimal scan interpretation and standardization, which is an essential step towards potential clinical implementation of ^89^Zr-immuno-PET.

For this study, we performed a multicenter interobserver analysis for data that was originally obtained in single center studies. With this experience, the next step towards standardization of quantification for ^89^Zr-immuno-PET studies can be done in the context of a multicenter study [e.g. the IMPACT trials, (NCT02228954, NCT02117466 and NCT01957332)].

Reliable delineation of tumor uptake on ^89^Zr-immuno-PET allows future use as a non-invasive clinical tool to determine mAb concentrations in the tumor. Knowledge on in-vivo drug delivery of mAb-based therapy (including antibody-drug conjugates, bispecific mAbs and immune checkpoint inhibitors) is crucial to understand and predict efficacy of treatment.

## Conclusion

This study shows that interobserver reproducibility of tumor uptake quantification on ^89^Zr-immuno-PET was excellent for SUV_max_ and SUV_peak_ using a standardized manual procedure for tumor segmentation. Semi-automatic delineation was not robust due to limited tumor contrast.

## Electronic supplementary material

Supplemental Table 1 (DOCX 14 kb)

Supplemental Table 2 (DOCX 14 kb)
