# Supplementary material for: Interobserver reproducibility of tumor uptake quantification with 89Zr-immuno-PET: a multicenter analysis
Source: Eur J Nucl Med Mol Imaging. 2019 Jun 17;46(9):1840–9. doi: 10.1007/s00259-019-04377-6 (PMC6647131; doi:10.1007/s00259-019-04377-6)
Supplement: Supplementary file 1 — (DOCX 14 kb) [file 259_2019_4377_MOESM1_ESM.docx]

**Supplemental Table 1. Interobserver variability for ^89^Zr-immuno-PET at D0**

|  | | ^89^Zr-rituximab | ^89^Zr-cetuximab |
| --- | --- | --- | --- |
|  |  | D0 | D0 |
| SUV_max_ | all | 19 (8-31);n=32  3.7 (2.4-5.3) | 6 (6-19);n=9  2.7 (2.2-5.3) |
|  | eligible | 17 (4-29);n=28  3.4 (2.2-5.3) | 8 (6-21);n=8  3.2 (2.5-5.4) |
|  |  |  |  |
| SUV_peak_ | all | 18 (9-30);n=32  3.2 (2.0-4.8) | 5 (4-18);n=9  2.5 (1.8-4.9) |
|  | eligible | 17 (8-24);n=28  3.0 (1.8-4.8) | 7 (4-21);n=8  3.0 (2.3-5.1) |
|  |  |  |  |
| SUV_mean_ | all | 13 (8-28);n=32  1.6 (1.3-2.3) | 10 (5-27);n=9  1.8 (1.2-3.1) |
|  | eligible | 12 (8-19);n=28  1.6 (1.3-2.3) | 17 (5-27);n=8  1.8 (1.7-3.2) |
|  |  |  |  |
| Volume  (mL) | all | 31(17-53);n=32  7.7 (4.4-19.1) | 33 (27-55);n=9  3.6 (2.2-9.9) |
|  | eligible | 26(16-45);n=28  7.7 (3.7-18.0) | 32 (26-49);n=8  3.2 (2.1-6.1) |
|  |  |  |  |
| TLU  (%ID) | all | 38(23-56);n=32  0.02(0.01-0.04) | 38 (33-63);n=9  0.01(0.01-0.02) |
|  | eligible | 34(20-46);n=28  0.01(0.01-0.04) | 38 (33-49);n=8  0.01(0.01-0.02) |
|  |  |  |  |

Data is presented as interobserver variability (CoV in %) on the first line and VOI metric on the second line (median value (interquartile range)).
