# Supplementary material for: Interobserver reproducibility of tumor uptake quantification with 89Zr-immuno-PET: a multicenter analysis
Source: Eur J Nucl Med Mol Imaging. 2019 Jun 17;46(9):1840–9. doi: 10.1007/s00259-019-04377-6 (PMC6647131; doi:10.1007/s00259-019-04377-6)
Supplement: Supplementary file 2 — (DOCX 14 kb) [file 259_2019_4377_MOESM2_ESM.docx]

**Supplemental Table 2 ICC calculations for all possible combinations of 2 observers**

| SUV_mean_ | ^89^Zr-rituximab | | | ^89^Zr-cetuximab | | | ^89^Zr-trastuzumab |
| --- | --- | --- | --- | --- | --- | --- | --- |
|  | D0 | D3 | D6 | D0 | D3 | D6 | D4 |
| O1, O2, O3 | 0.77  (0.62-0.87) | 0.90  (0.80-0.96) | 0.94  (0.90-0.97) | 0.77  (0.41-0.95) | NA | 0.92  (0.73-0.98) | 0.93  (0.86-0.97) |
| O1, O2 | 0.79  (0.60-0.90) | 0.86  (0.60-0.95) | 0.96  (0.91-0.98) | 0.94  (0.73-0.99) | NA | 0.96  (0.75-0.99) | 0.94  (0.87-0.98) |
| O1, O3 | 0.83  (0.65-0.92) | 0.94  (0.75-0.98) | 0.94  (0.88-0.97) | 0.61  (-0.05-0.92) | NA | 0.88  (0.28-0.98) | 0.93  (0.83-0.97) |
| O2, O3 | 0.69  (0.44-0.85) | 0.91  (0.80-0.96) | 0.93  (0.86-0.97) | 0.70  (0.07-0.94) | NA | 0.93  (0.71-0.99) | 0.93  (0.83-0.97) |

Data presented as ICC (95% confidence interval).

All ICC calculations were performed on VOI eligible for quantification (n=84).

D0 VOI were delineated on D6 and imported to the D0 scan (data marked in grey).
